# The Environment Shapes the Inner Vestibule of LeuT

**DOI:** 10.1371/journal.pcbi.1005197

**Published:** 2016-11-11

**Authors:** Azmat Sohail, Kumaresan Jayaraman, Santhoshkannan Venkatesan, Kamil Gotfryd, Markus Daerr, Ulrik Gether, Claus J. Loland, Klaus T. Wanner, Michael Freissmuth, Harald H. Sitte, Walter Sandtner, Thomas Stockner

**Affiliations:** 1 Medical University of Vienna, Center for Physiology and Pharmacology, Institute of Pharmacology, Vienna, Austria; 2 University of Copenhagen, Faculty of Health and Medical Sciences Denmark, Department of Neuroscience and Pharmacology, Copenhagen, Denmark; 3 University of Copenhagen, Faculty of Health Sciences Denmark, Department of Biomedical Sciences, Copenhagen, Denmark; 4 Ludwig Maximilians University Munich, Department of Pharmacy, Center of Drug Research, Munich, Germany; Icahn School of Medicine at Mount Sinai, UNITED STATES

## Abstract

Human neurotransmitter transporters are found in the nervous system terminating synaptic signals by rapid removal of neurotransmitter molecules from the synaptic cleft. The homologous transporter LeuT, found in *Aquifex aeolicus*, was crystallized in different conformations. Here, we investigated the inward-open state of LeuT. We compared LeuT in membranes and micelles using molecular dynamics simulations and lanthanide-based resonance energy transfer (LRET). Simulations of micelle-solubilized LeuT revealed a stable and widely open inward-facing conformation. However, this conformation was unstable in a membrane environment. The helix dipole and the charged amino acid of the first transmembrane helix (TM1A) partitioned out of the hydrophobic membrane core. Free energy calculations showed that movement of TM1A by 0.30 nm was driven by a free energy difference of ~15 kJ/mol. Distance measurements by LRET showed TM1A movements, consistent with the simulations, confirming a substantially different inward-open conformation in lipid bilayer from that inferred from the crystal structure.

## Author Summary

Crystal structures of the bacterial small amino acid transporter LeuT provided structural evidence for the alternating access model. Thereby, these structures shaped our understanding of the mechanisms underlying substrate translocation by neurotransmitter transporters. However, it has been questioned, if the crystallized inward-open conformation of LeuT can exist in the membrane environment. Here we show that, while stable in detergent micelles, the inward-open conformation of LeuT is of high energy and undergoes structural readjustments. We use a multi-faceted approach including molecular dynamics simulations, scintillation proximity assays, free energy calculations and apply for the first time lanthanide resonance energy transfer measurements to verify the *in silico* predictions. *In silico* and *in vitro* approaches using the same conditions allowed us to combine the macroscopic experimental data with microscopic all atom results from simulations to identify the underlying driving forces: partitioning of charged and polar groups from the hydrophobic membrane interior to the hydrophilic environment. We propose that the inward-facing state shows a much smaller movement of TM1A, but large enough to create an access path to the S1 substrate binding site from the vestibule.

## Introduction

Stringent regulation of neurotransmission is essential for brain function. Secondary active transporters from the solute carrier family 6 (SLC6) terminate signal propagation by clearing released neurotransmitters from the synaptic cleft. This is achieved by coupling substrate transport to the electrochemical sodium gradient. Members of the SLC6 family include the transporters for dopamine (DAT), norepinephrine (NET), serotonin (SERT) and γ-aminobutyric acid (GAT). Several neurological and psychiatric disorders are associated with SLC6 dysfunction [1]. In addition, these transporters are clinically relevant targets of a number of marketed drugs but also of illicit drugs of abuse.

The crystal structures of the bacterial homolog neutral amino acid transporter (LeuT) from *Aquifex aeolicus* in three different conformations [2–4] have greatly enhanced our structural but also functional understanding of the SLC6 family. More recently, the crystal structures of the *Drosophila melanogaster* DAT [5–7] confirmed fold conservation. Importantly, many insights gained from the LeuT structures can be extrapolated to eukaryotic SLC6 members. Overall, SLC6 transporters consist of twelve transmembrane helices (TM), which are assembled into two domains. The structures imply that the scaffold domain anchors the transporter into the membrane, while the bundle domain changes its conformation to afford substrate transport [8, 9]. Substrate and two sodium ions bind to the substrate binding site (S1) located in the center of the membrane [3]. The structural dynamics of LeuT have been directly measured by fluorescence resonance energy transfer microscopy (FRET) [10–13], electron paramagnetic resonance (EPR) [14–17], and investigated by molecular dynamics (MD) simulations [12, 17–37]. These studies revealed that substrate transport by LeuT involves multiple conformations and that the structure of LeuT is highly dynamic. However, it still remains elusive, if the first part of TM1 (TM1A) can detach from the bundle domain to allow for substrate release [2, 8, 21, 22]. The inward-open LeuT crystal structure reveals a 45° rotation of TM1A, thereby opening a large access path to the S1 site from the cytosol [2]. The structure was stabilized by an antibody and four mutations were introduced. Importantly, if this motion occurred, TM1A would partition into the hydrophobic core of the membrane and, as a consequence, hydrophilic and charged residues would be transferred into the membrane, which is an energetically unfavorable process.

In the current study we investigated the conformation of the inward-open state in a detergent micelle and in a membrane bilayer to assess the impact of the environment on the shape of the inner vestibule. MD simulations revealed that the conformation adopted by LeuT in the crystal structure is stable in a micellar environment. In contrast, the polar and charged residues leave the hydrophobic core of the lipid bilayer and partition in the hydrophilic environment of the lipid head groups and bulk water. We confirmed our *in silico* results by experimental measurements using lanthanide resonance energy transfer (LRET) [38, 39]. We observed changes in distances between the micelle and the POPC membrane environment that were consistent with the results from MD simulations. We deduced from our data that the conformation of TM1A in the inward-open state depends highly on its immediate environment and inferred that the freedom of movement is considerably smaller in a cell membrane than suggested from crystal structures obtained in micellar systems.

## Results

### Structural stability of the inward-open transporter

We investigated the influence of the environment on the conformation of the inward-open LeuT structure by MD simulations. We inserted the inward-open structure of wild type LeuT into (i) n-octyl-beta-D-glucoside (BOG) to mimic the micellar environment used during the crystallization process and into (ii) 1-palmitoyl-2-oleoyl-sn-glycero-3-phosphocholine (POPC) to resemble the cell membrane phospholipid bilayer environment. In addition, the outward-occluded conformation was simulated in a POPC membrane and used as reference. All MD simulations were carried out in complex with sodium ions and the substrate leucine. The outward-occluded LeuT conformation showed a high overall stability in the membrane environment in repeated MD simulations of 200 ns length. Root mean square deviations (RMSD) from the starting structure were below 0.20 nm (S1 Fig).

**Fig 1:**
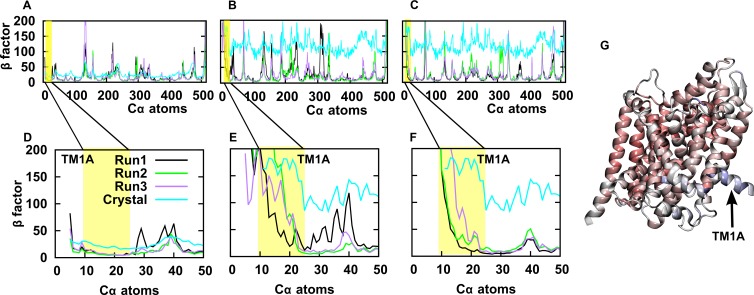
Comparison of residue mobility (β-factor). Comparison of β-factors extracted from crystal structures (outward-occluded: 2A65; inward-open: 3TT3) with β-factors calculated from MD simulations for **(A)** the membrane inserted outward-occluded LeuT, **(B)** membrane inserted inward-open LeuT and **(C)** micelle inserted inward-open LeuT. **(D-F)** Zoom into the region of TM1A (highlighted in yellow). The color code of black, green, purple and cyan is used throughout the manuscript to identify respectively run 1, 2, 3 and the crystal structure. **(G)** The structure of the inward-open LeuT is color coded (from red to blue) by β-factors as reported in the crystal structure (PDB ID: 3TT3).

Atomic β-factors reflect the uncertainty in the determination of atomic positions; in crystallography, these are often interpreted as mobility and related to dynamic properties of the corresponding residue. A comparison of the β-factors calculated from root mean square fluctuations in our MD simulations with the β-factors obtained from the outward-occluded crystal structure (PDB ID: 2A65; resolution: 1.65 Å) [3] revealed comparable profiles as observed in previous studies [18], (Fig 1A). Also, MD simulations of the membrane inserted inward-open LeuT showed a similar β-factor profile (Fig 1B and 1C), though the amplitudes of the motions were larger (Fig 1A and 1D). The crystal structure of the inward-open conformation (PDB ID: 3TT3; resolution: 3.22 Å) [2] had a much lower resolution, β-factors are therefore higher (Fig 1B and 1C).

The crystal structures of LeuT were solved in the presence of BOG or BSG (n-octyl-beta-D-thioglucoside), but the detergent molecules were largely unresolved. Hence, we developed a detergent solubilized LeuT system by applying a self-assembly procedure. We tested three detergent:transporter ratios: 120:1, 140:1 and 160:1, as the exact detergent:transporter ratio is unknown. The 120:1 ratio was likely too low, because the membrane exposed hydrophobic surface of LeuT was not completely covered with BOG molecules. The hydrophobic surface was fully covered at a 140:1 detergent:transporter ratio. At a 160:1 detergent-transporter ratio we observed detergent-only micelle formation suggesting that this ratio might be too high. We therefore selected the 140:1 detergent:transporter ratio for all subsequent simulations. A recent study found that a similar number of n-dodecyl-β-D-maltopyranoside (DDM) detergent molecules was needed for solubilization of LeuT [34]. Structural stability in MD simulations of the micelle inserted LeuT was higher than in the membrane environment (Fig 1C), but the β-factors showed the same pattern at lower amplitudes (Fig 1F).

### Mobility of TM1A

The crystal structure of LeuT in the outward-occluded conformation has been solved starting from residue 5 (PDB ID: 2A65). Notably, the first 10 residues were missing in the inward-facing crystal structure (PDB ID: 3TT3), suggesting that their positional uncertainty was too high. The β-factors of TM1A did not differ from the other TM helices in the outward-occluded crystal structures (Fig 1E), while in the inward-facing structure, the β-factors of TM1A were significantly higher (Fig 1G). This suggests higher mobility or the existence of multiple conformational states of TM1A. MD simulations showed similar results: the mobility of TM1A was low in the outward-occluded conformation (Fig 1D) and comparable to that of the other TM helices. In contrast, in the inward-open conformation, the β-factors of TM1A were also much higher than those of the other TM helices, in particular in the membrane environment (Fig 1E and 1F). Furthermore, residues preceding TM1A showed essentially unconstrained movement. The mobility determined in MD simulations was therefore consistent with the β-factors measured in the crystal structures. Importantly, this analysis showed that, in the inward-open conformation, the state of TM1A differed substantially from that seen in the outward-occluded conformation.

**Fig 2:**
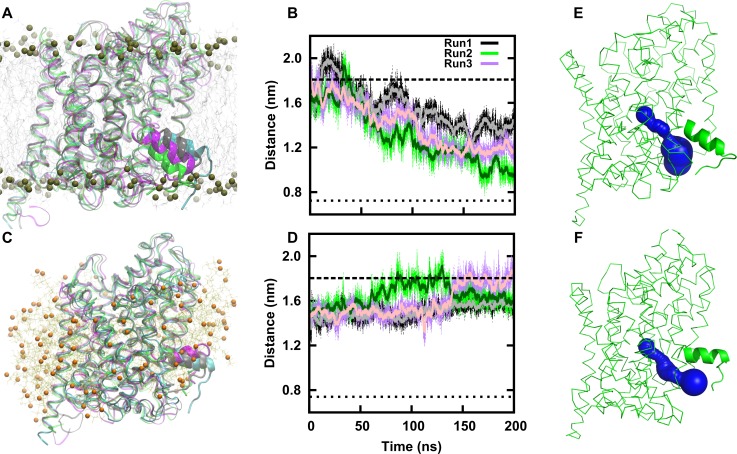
Dynamics of helix TM1A. **(A)** Comparison of the final structures of three independent simulations (grey, green, purple) of membrane-inserted LeuT with the inward-open crystal structure (PDB ID: 3TT3) shown in cyan. Lipid molecules are shown in grey, the dark spheres represent the phosphate atoms of the membrane lipids. **(B)** Change in vestibule size of membrane inserted LeuT over time is quantified by measuring the distance between the Cα of residue M18 (TM1A) and Y265 (TM6). The values of each time frame (thin line) are shown together with a running average over 100 frames (thick line). The distances as observed in the crystal structure of LeuT (dashed line for the inward-open structure with the PDB ID: 3TT3; a dotted line for the outward-occluded structure with the PDB ID: 2A65) are also shown. **(C)** Comparison of the final structures of three independent simulations (grey, green, purple) of micelle-inserted LeuT with the crystal structure (PDB ID: 3TT3) shown in cyan. Detergent BOG molecules are shown in yellow, atom O1 as orange spheres. **(D)** Change in vestibule size as described in B for detergent solubilized LeuT. **(E, F)** The size of the vestibule is shown for the final conformation of run 2 of membrane-inserted LeuT **(E)** and of run 2 of micelle-inserted LeuT **(F)**. The protein is shown in green, the pore surface in blue, as calculated by the program caver 3.0.

TM1A was deeply buried in the hydrophobic core of the membrane bilayer in the starting structure of the MD simulations of the inward-open conformation. We observed a directional movement of TM1A during MD simulations, whereby polar and charged moieties of TM1A (consisting of the side chain of R11 and the backbone amide groups on the first helical turn of TM1A (residue 10 to 13)) partitioned out of the hydrophobic core of the membrane (Fig 2). This transition was accompanied by a change in size of the inner vestibule, which decreased in width over time. We determined the distance between the Cα atoms of residues M18 (TM1A) and residue Y265 (TM6; Fig 2B) across the inner vestibule as a parameter to estimate its width. The distance decreased from 1.7 nm to 1.2–1.4 nm in two independent MD simulations. The third MD simulation showed a drop in distance to 0.9 nm. Despite the large movement, the inner vestibule did not completely close (Fig 2E and S2 Fig).

MD simulations of the micelle-inserted inward-open LeuT showed a strikingly different motion, when compared to the membrane embedded state: movements of TM1A were non-directional and β-factors showed larger positional fluctuations. The final conformations of three MD simulations overlapped with each other and with the crystal structure (Fig 2C). The inner vestibule diameter did not decrease over time and accordingly the access path to the S1 site did not narrow significantly (Figs 2D, 2F and S2). These observations imply that the mobility of TM1A is high, presumably because it is less constrained by interactions with the other TM helices than in the outward occluded state.

### The membrane restraints the movement of TM1A

**Fig 3:**
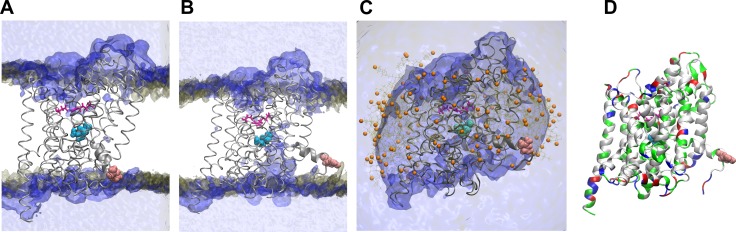
Water density in LeuT. Water (blue) and phosphate (of POPC lipids) density (brown) were averaged over the 200 ns of run 1. The starting structure of LeuT is shown, the substrate leucine (cyan), the salt bridge of the outer vestibule (magenta), R11 (pink) and the O1 atoms of BOG (orange) are highlighted. **(A)** Water penetrates until the salt bridge of the extracellular gate (R30 and D404) in the outward-occluded state. **(B)** Opening of the inner vestibule allowed water to reach the substrate of the inward-open LeuT. **(C)** Similar water penetration was observed in the micelle embedded inward-open LeuT. (**D**) The physico-chemical properties of all residues are mapped onto the structure of the inward-facing structure using a color code: non-polar (white), polar (green), positively charged (blue), negatively charged (red).

A fundamental difference between the outward- and the inward-facing conformations of LeuT lies in the access path to the central binding site. To clarify these changes, we derived water density plots from our MD simulation on the inward-open and outward-open states. In the case of the outward-open state, water penetrates from the extracellular space into the substrate-binding site S1 (Fig 3 and 3A). In simulations of the outward-occluded state, we observed that the S1 site is sealed off from the extracellular space by a thin gate. This gate is formed by a salt bridge between residues R30 and D404 (colored magenta in Fig 3) and the hydrophobic lid comprised of the residues, Y108 and F253. In the inward-open conformation, we observed that water reaches the S1 binding site from the cytosol (Fig 3B). This difference reflects the main conformational change in the transport cycle. The thin outer gate is strengthened by movements of the bundle domain which leads to a broadening of the water-free region in the outer vestibule [2, 3].

Fig 3B shows that the charged side chain of R11 (colored pink in Fig 3) is located within the hydrophobic membrane core in the starting conformation. Residue R11 increased interactions with the hydrophilic environment over time, mainly with the negatively charged PO_4_^-^ group of the membrane lipids and with water (S3 Fig). Concomitantly, the first turn of helix TM1A partitioned into the polar environment of the lipid head group region. The first turn of any helix establishes a strong helical dipole due to the alignment of the four consecutive backbone amide protons (residue 10 to 13). Increasing interactions with the polar environment correlated with the movement of TM1A. BOG detergent molecules cover the hydrophobic transmembrane region of LeuT and formed a thin donut-shaped structure around the hydrophobic surface of LeuT in the micelle system (Fig 3C). The spherical shape and the small width of the BOG micelle structure allow for hydration of R11 and the backbone amide groups of TM1A. LeuT does not show a precisely match with the hydrophobic core of the membrane [31]. The hydrophobic mismatch is not evenly distributed over the membrane-protein interface. This is in accordance with our finding that insertion of TM1A into the membrane core further distorts the membrane and affected membrane thickness (S4 Fig). Membrane deformation relaxed towards the end of the MD simulation, particularly after re-partitioning of TM1A.

### The protein conformation determines the stability of the bound substrate and sodium ions

**Fig 4:**
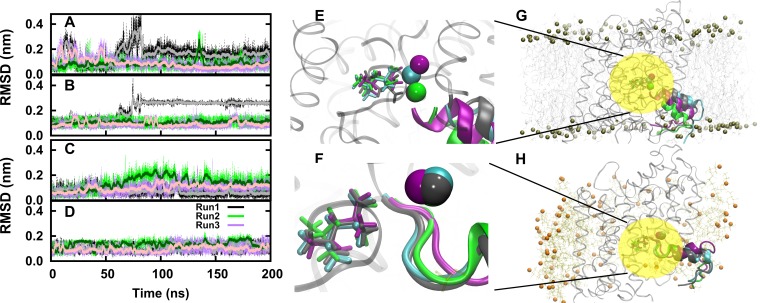
Movement of sodium 1 and substrate of the inward-open conformation. Panels **A, B** show the displacement of sodium 1 and substrate from three independent simulations of the membrane inserted inward-open LeuT. Panels **C, D** show the respective movement of the micelle embedded LeuT. **Panels E-H**: The final structures of three independent simulations of 200 ns duration each are shown along with the starting structure for the membrane inserted LeuT in cyan (**E, G**) and micelle embedded (**F, H**). Please note, the final positions of Na1 are essentially identical in two (green and gray) simulations in panel E.

Substrate and two sodium ions are stably bound to the S1 site in the outward-occluded state. Their mobility was very low: the RMSD remained below 0.1 nm. The situation was different in the inward-open conformation (Fig 4): MD simulations of the membrane inserted inward-open LeuT suggested that this state represented a conformation, in which substrate and the two sodium ions were prone to release into the cytosol. The sodium in the Na2 site became hydrated and dissociated into the bulk solvent through the open inner vestibule within the first nanoseconds (S5 Fig) similar to recent reports [36]. This was expected, because the Na2 site is disrupted in the inward-open crystal structure due to the movement of the bundle domain and mutations at the Na2 binding site [2]. Ion binding to and the selectivity of the Na2 site is very sensitive to the local environment, which consists of residues from TM1 and TM8 [19, 26].

The extent of Na1 and substrate hydration was higher in all MD simulations of the inward-open conformation as compared to the level of hydration in the outward-occluded conformation. Importantly, substrate and Na1 were mobile in all inward-open simulations (Fig 4). Yet, neither Na1 nor the substrate left the vestibule in 200 ns simulation runs. The most likely reason is a too short simulation. Nonetheless, we interpret our simulations as showing the initial events of substrate release into the cytosol.

### Experimental determination of the conformation of TM1A

**Fig 5:**
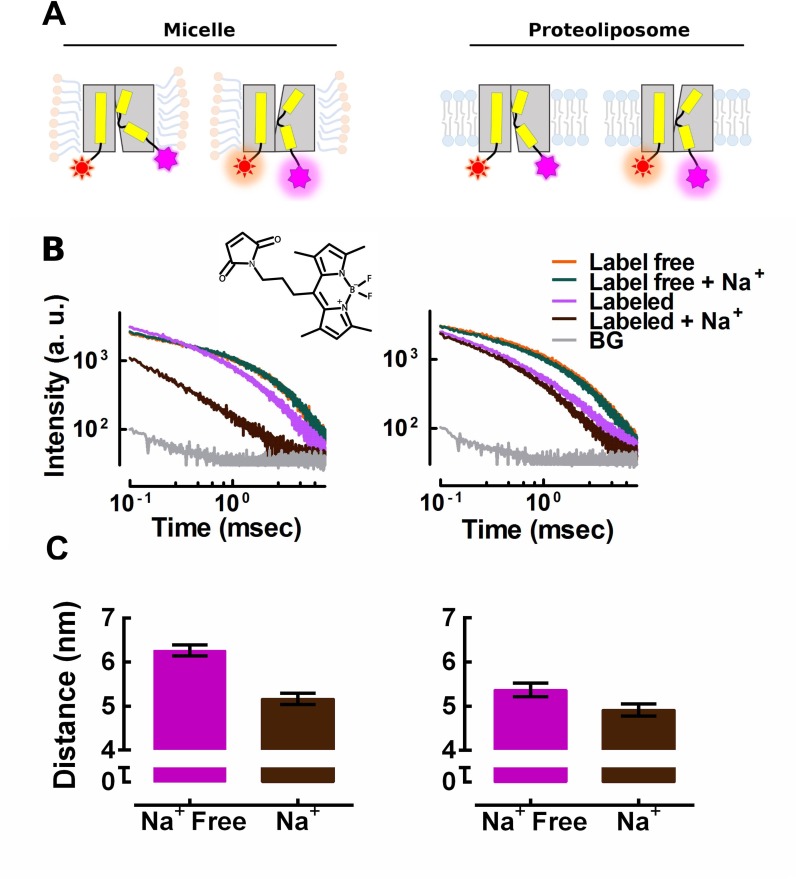
LRET based distance measurements. **(A)** Schematic rendering of the LRET experiment. The Tb^+3^donor is in complex with the LBT tag (red) inserted at the C-terminus; the BODIPY-C3-M acceptor dye (magenta) is chemically linked to residue 9 at the N-cap motif TM1A. **(B)** Representative Tb^+3^ decay traces either incorporated into micelles or reconstituted into proteoliposomes, either unlabeled or labeled with the BODIPY-C3-M acceptor dye, in the presence of 200 mM Na^+^ or Na^+^ free. The structure of BODIPY-C3-M is shown in the insert. **(C)** Distances calculated from the donor decay between the Tb^+3^ and the acceptor dye. Shown are means ± S.E.M. from three independent experiments done in triplicate.

The computational analysis predicted that the conformation of TM1A of the inward-open LeuT differed between the detergent solubilized and the lipid bilayer environment. We employed lanthanide-based resonance energy transfer (LRET) to verify the predictions by measuring intramolecular distances. LRET offers the advantage over conventional fluorescent energy transfer (FRET) measurements that emission is isotropic. This renders energy transfer much less sensitive to the orientation of the label [38]. We introduced a lanthanide binding tag (LBT) and used the Tb^3+^ ion as donor. The LBT tag chelates Tb^3+^ and protects it from relaxation by direct water contacts. Lanthanides have a low extinction coefficient and *per se*, cannot be efficiently excited. However, the LBT carries a tryptophan residue, which serves as sensitizer antenna and transfers the initial excitation energy to the chelated Tb^3+^ ion. The LBT tag was inserted at the C-terminal end of LeuT. We synthesized the fluorophore BODIPY-C3-M (Fig 5, see Materials and methods for the synthesis details), which gives rise to reduced positional uncertainty in LRET, because of (i) the symmetric attachment and (ii) the very short linker (0.3 nm) between the BODIPY dye and the thiol reactive maleimide function. The BODIPY-based acceptor fluorophore was covalently linked to a cysteine introduced at position 9 (instead of alanine) of the otherwise cysteine-free LeuT. This position was selected because it is juxtaposed to the N-cap of TM1A and lies on the opposite side of the C-terminus across the vestibule. The resulting double-tagged construct is referred to as LeuT-LBT-A9C in the subsequent description. Insertion of the donor and acceptor probes did neither alter substrate affinity of nor maximum velocity of transport by LeuT-LBT-A9C (S6 Fig) compared to WT.

The LRET experiment is schematically depicted in Fig 5A. The LBT chelated Tb^3+^ donor is shown in red, the acceptor fluorophore bound to A9C is shown in magenta. Movements of TM1A can be detected by a change in the donor-acceptor distance. The LeuT-LBT-A9C construct was solubilized in n-dodecyl-β-D-maltoside (DDM) detergent micelles or reconstituted into POPC to generate proteoliposomes. Fig 5B shows recordings of LRET signals emitted from the Tb^3+^ donor after excitation by a brief laser pulse of 30 ns length. Traces of label free LeuT-LBT-A9C (orange and green) show donor emission in the absence of the BODIPY-C3-M acceptor. Recordings in the presence of BODIPY-C3-M were measured in the absence (purple) or presence of 200 mM Na^+^ (black). The donor decay was faster in the presence of the acceptor because of quenching by energy transfer (S1 Table). The measured decay constant was converted to the corresponding donor-acceptor distance by the Förster equation. The grey traces represent the background recorded with wild type LeuT lacking the LBT tag. The ionic composition did not affect donor emission, because the decay rates were similar in the presence and absence of Na^+^ (replaced by K^+^).

We measured slower decay rates in the absence of Na^+^ than in its presence. Thus donor and acceptor were in closer proximity in the presence of Na^+^. This is consistent with the results from previous studies [10–12, 15], which applied single molecule FRET to detergent solubilized LeuT. In the micelle environment, the distances were 6.26 ± 0.21 nm and 5.16 ± 0.22 nm in the absence and presence of 200 mM Na^+^, respectively. In contrast, the distance decreased from 5.34 ± 0.15 nm in the absence of Na^+^ to 4.80 ± 0.14 nm in its presence, if the recordings were done with membrane embedded LeuT-LBT-A9C. Thus, the conformational change induced by Na^+^ reduced the distance by only 0.54 ± 0.21 nm in the membrane environment of proteoliposomes but by 1.11 ± 0.30 nm in the detergent-solubilized transporter.

### Potential of mean force of TM1A partitioning

**Fig 6:**
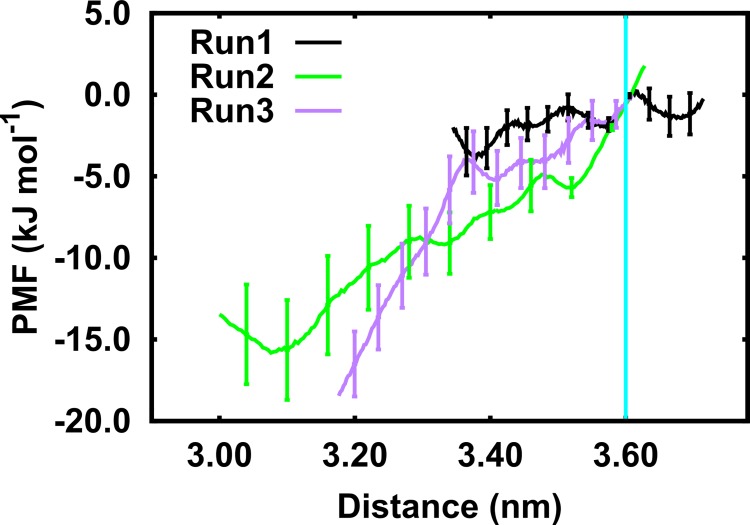
Potential of Mean Force (PMF) of TM1A movement. The center of mass distance between the Cα atoms of TM1A (residues 11 to 20) and the Cα atoms of IL1 (residues 78–81), TM8 (residues 363–366) and TM12 (residues 503–504) was used as reaction coordinate. The PMFs were constructed using umbrella sampling combined with the weighted histogram analysis method, error bars were estimated by applying the bootstrap method. For simplicity, only every ~15^th^ error bar from the bootstrap results is shown. The distance measured in the inward-open crystal structure is shown as vertical cyan line.

The simulations showed that TM1A partitioned out of the hydrophobic core of the membrane (Fig 2) and this was confirmed by distance measurements (Fig 5). We performed potential of mean force (PMF) calculations to quantify the free energy profile underlying this movement. We extracted equally spaced (0.02 nm) conformations from the three inward-open membrane-inserted simulations and performed 25 ns long umbrella sampling calculations for each window. We excluded the first 5 ns as equilibration and construct the PMF using the weighted histogram analysis method (WHAM) (Fig 6) using the remaining 20 ns. Statistical errors were estimated using the bootstrap method.

The profiles showed that re-partitioning of TM1A is an energetically favorable process. The initial conformation was less stable by approximately 15 kJ/mol than the lowest energy conformation. The final rise of the PMF profile could indicate that TM1A reached an equilibrium position. The profile of run 1 remained approximately flat, in line with the minimum TM1A movement in the equilibrium simulation and the largest membrane deformation (S4 Fig). The intrinsically slow membrane dynamics therefore did not allow for reaching full convergence within the 200 ns of the equilibrium MD simulations. The free energy profiles show a clear trend towards an energetically more stable conformation in which the charged and polar moieties of TM1A have partitioned into the polar environment of the head group region of the membrane.

We infer from the equilibrium simulations and from the PMF free energy profile that the extent of TM1A movement in the inward-open state is much smaller in the membrane environment than that suggested by the crystal structures [2, 3]. However, this conformation provides an open access path to the S1 binding site.

## Discussion

The neutral amino acid transporter LeuT translocates substrates by an alternating access mechanism, in which the central binding site is only accessible from one side of the membrane at any given time [2–4, 8, 21, 40, 41]. The outward-open [4] and inward-open [2] structures are thought to represent endpoints of the transport cycle. TM1A showed high β-factors in the inward-open conformation, while the electron density of the 10 N-terminal residues could not be resolved. The inward-open conformation of TM1A observed by crystallographically has remained controversial, because of its questionable compatibility with the physico- chemical constraints imposed by the membrane bilayer. The mutations that needed to be introduced to obtain the inward-facing conformation added to the controversy, because of the disruption of the Na2 sodium binding site. Our results show that the crystallographically observed conformation of TM1A can be accommodated by a micelle environment, but it does not occur in a membrane. This conclusion is based on the following observations: (i) we found a stable conformation and detected a large mobility of TM1A in BOG micelle simulations, which is in line with the β-factors reported in the crystal structure. The structure was stable because detergent molecules covered the entire membrane exposed surface of LeuT. A similar behavior was observed with the detergent DDM [34]. Importantly, the micelle structure allowed for hydration of the charged side chain of R11 and of the dipole created by the first turn of the helix backbone. (ii) In detergent, TM1A showed high mobility, because it was not constrained by any large interaction surface. (iii) Our simulations revealed that the conformation of TM1A was not compatible with a membrane environment. In fact, the hydrophobic effect exerted by the lipids and the electrostatic interactions of TM1A destabilized the starting conformation and induced conformational rearrangements. (iv) We confirmed earlier findings [31] that the proposed inward-open conformation of LeuT caused membrane deformation. The movement of TM1A, which we observed, reduced this hydrophobic mismatch.

We verified our conclusion using LRET-based distance measurements: for the inward-open conformation of LeuT, the MD simulations of the micelle-covered LeuT predicted a distance between the Cα atoms of the N-terminal residue 9 and Cα of residue 508 at the C-terminus of TM12 of 4.81 ± 0.25 nm. Complete partitioning of TM1A was observed in two simulations of membrane-embedded LeuT and reduced the distance to 4.51 ± 0.18 nm. The corresponding changes in distance measured by LRET between outward- and inward-facing LeuT were 1.11 ± 0.30 nm and 0.54 ± 0.21 nm for the micelle and membrane environment, respectively. Thus, upon switching from the outward-facing sodium-bound state of LeuT, TM1A was displaced to a lesser extent, if the protein resided in the membrane. The measured difference in movement between micelle-covered and membrane-embedded LeuT (0.56 ± 0.37 nm) is in excellent agreement with the predictions from the MD simulations (0.30 ± 0.30 nm). The fact the LRET-measurements indicate a more extensive repositioning of TM1A can be rationalized by considering the applied rulers, i.e. the Cα atoms of the protein backbone (in the MD-simulations) and the fluorescent probes, which were–by necessity–further apart. We are aware that our simulations did not explicitly include the position of the LBT and the mobility of the acceptor BODIPY-C3-M (resulting from the short flexible linker). However, the direct comparison between experiments and MD simulations is justified under the following assumptions: (i) the vector connecting donor and acceptor in the LRET experiment and the vector connecting the Cα atoms of residue 9 and 508 in the MD simulations are essentially parallel and/or their relative angle does not change substantially between inward and outward-facing states. (ii) The conformational ensembles of the LBD tag and of the BODIPY-C3-M acceptor dye remain unchanged during the switch from outward to inward-facing state. These conditions ought to be met in our system. First, the LBT tag is attached to the scaffolding domain, which does not undergo any motion during the conformational switch. Second, the acceptor is attached to residue 9, thus both the Cα of this residue and the attached fluorescent tag move in concert. We can therefore infer from our MD simulations and our experiments that the movement of TM1A is much smaller in the membrane environment than that suggested by the crystal structures. Importantly, the exit path from the S1 site to the cytosol remained open, indicating that the large amplitude of the TM1A motion is not a requirement for substrate release.

We used PMF calculations to quantify the free energy profile of TM1A movement starting from conformations extracted from equilibrium MD simulations, in which the system was allowed to explore the available phase space in an unbiased fashion and to move down naturally existing energy gradients. We found that the crystal structure was in a high-energy conformation within the membrane environment and found that partitioning of TM1A released ~15 kJ/mol. The conformation of TM1A has been investigated in two recent simulation reports. TM1A was shown to have a high probability to partially close the inner vestibule in a 1-palmitoyl-2-oleyl-phosphatidylethanolamine (POPE) membrane [42]. The opposing result was reported earlier, as TM1A was found to be very stable when residing in the hydrophobic core of a POPC membrane (stabilized by ~20 kJ/mol) [36]. However, in spite of this net stabilization energy calculated from the PMF profile, that study nevertheless suggested that TM1A could deviate from the crystallized conformation [36].

The orientation of TM1A has also been studied by single molecule FRET [11] and EPR in the micellar environments [14] using a label attached to residue 7. Both studies reported that LeuT only populated the outward-facing state in the presence of sodium or sodium and substrate, while in the absence of sodium and substrate two conformations were found, which are consistent with inward and outward-facing LeuT. In contrast, only one distance was detected when the polar EPR label was directly attached to TM1A to the membrane exposed residue 12 [14]. Here we report experimental distance measurements in micelles and we extend the experimental investigation to a physiologically more relevant POPC membrane environment. To emphasize the importance of the environment, we observed substantial environmental effects: (i) we find in the micelle environment that TM1A maintains a conformation consistent with the crystal conformation. (ii) In contrast, in the lipid environment TM1A moves closer to the C-terminus, thereby reducing the opening of the inner vestibule. Thus, both the experimental results and the data from simulations indicate that TM1A does not fully enter the hydrophobic core of the membrane, but differs from the conformation in the outward-facing state.

Movements of helix TM1A disrupt the geometry of the Na2 binding site, because it is formed by TM1 and TM8, which move relative to each other [2, 3, 8, 13, 22]. The implication of our observations is that once TM1A moves, the Na2 binding site is disrupted, which leads to rapid dissociation of Na2 [13]. We observed repeated spontaneous movement events of Na1 and substrate after dissociation of Na2, implying that the affinity for sodium and substrate is low in the inward-open state.

LeuT has served as a template for the SLC6 transporter family since its first crystallization. It was repeatedly demonstrated that many insights gained from LeuT were also relevant to understanding the transport cycle of the human neurotransmitter transporters. It is hence very likely that our findings can also be generalized, because the biophysical properties of the environment impose similar constraints on all transporters: the detergent micelles can adjust to any shape of the solubilized membrane protein, but the two dimensional layer of the lipid bilayer environment opposes motion against the pressure profile [43–45] and precludes vertical movements of helices, *i*.*e*. in a direction perpendicular to the plane of the membrane. It can therefore be safely assumed that movements of TM1A in the human SLC6 transporter family will be restrained in amplitude by the membrane environment.

## Materials and methods

### Model building

Simulations of the inward-facing structure of LeuT (PDB ID: 3TT3) [2] were carried out in palmitoyl-oleoyl-phosphatidyl-choline (POPC) containing membranes and in n-octyl-β-D-glucopyranoside (BOG) micelles. The missing residues of the inward facing LeuT structure were built using MODELLER, version 9.12 [46] applying the automodel procedure, selecting the best models according to the DOPE score [47]. Mutations present in the structure were reverted to wild type. The missing residues of the N-terminus were copied from the outward-occluded LeuT structure (PDB ID: 2A65) [3] after superpositioning of helix TM1A, while the sodium ions and the substrate leucine were copied after superpositioning of the scaffold domain. Residues E112, E287 and E419 are protonated as suggested by crystal structures and pKa calculations [8]. The side chain of residue K288 in the center of the membrane was neutralized. The LeuT structure with the PDB ID: 2A65 was used as starting structure for the simulations of the outward-occluded structure. The missing 3 residues of the extracellular loop 2 were modeled using MODELLER. The transporter structures were inserted into a pre-equilibrated membrane consisting of 174 POPC lipid molecules using the membed method as described recently [48]. Each system contained ~12850 SPC water molecules, was neutralized and a salt concentration of 150 mM NaCl was added by randomly replacing water molecules. The system dimensions are 8.37, 8.37 and 9.70 nm. The semi-isotropic pressure coupling scheme was applied. Each system was equilibrated for a total of 30 ns while slowly releasing the transporter by reducing the position restraints on the heavy atoms of LeuT in three steps: 1000, 100 and 10 kJ/mol/nm^2^. Representative structures are shown in S7 Fig.

### Micelle formation

The micelle structure was created using a self-assembly procedure. We randomly placed BOG detergent molecules into a truncated octehedron box that contained one LeuT using the same starting conformation as in the membrane simulations and filled the system with ~48000 SPC water molecules. The systems were electroneutralized and an ionic concentration of 150 mM NaCl was added by randomly replacing water molecules. Electrostatic interactions were treated using the particle mesh Ewald summation method with a cutoff of 1.0 nm. The heavy atoms of LeuT were restrained with 1000 kJ/mol/nm^2^. These assembled systems were further equilibrated for 10 ns while maintaining secondary structure using distance restraints. The BOG detergent molecules were allowed to equilibrate for 100 ns. We built independently three systems by incrementally adding BOG detergent molecules. The protein:BOG ratios were 1:120, 1:140 and 1:160. The 120 BOG detergent molecules were not sufficient to fully cover the hydrophobic region of LeuT. Addition of 20 randomly placed BOG molecules to the equilibrated first systems (ratio 1:140) followed by 100 ns equilibration allowed for a good coverage of the hydrophobic surface. Addition of further 20 detergent molecules (ratio of 1:160) resulted in the formation of small detergent micelles suggesting that the lipid:protein ratio was too high. Representative structures of the 1:140 system are shown in S7 Fig.

### Simulation parameters

Production runs were carried out for 200 ns. Simulations were carried out with the Gromacs simulations package, version 4.5.4 [49]. Berger lipids [50] were used for describing the POPC membrane. The OPLS force field [51] was used for the protein and detergent. The topology for the BOG detergent molecule was developed using the mktop procedure applying the standard OPLS partial charge scheme. Temperature was maintained at 310 K using the v-rescale (τ = 0.1 ps) thermostat [52], while separately coupling protein, membrane and solvent. Pressure was maintained at 1 bar using the Berendsen barostat [53]. The pressure coupling constant was set to 1.0 ps, the compressibility to 4.5×10^−5^ bar^-1^. Long range electrostatic interactions were described using the smooth particle mesh Ewald method [54] applying a cutoff of 1.0 nm. The van der Waals interactions were described using the Lennard Jones potential applying a cutoff of 1.0 nm. Long range correction for energy and pressure were applied. The bonds and angles of the water molecules were constrained using the SETTLE algorithm [55], while all other bonds were constrained by LINCS [56].

### Potential of mean force calculations

We used potential of mean force (PMF) calculations [57] to quantify the energetic profile for the movement helix TM1A. Structures were extracted from the equilibrium simulations in 0.02 nm increments. Each structure was used as starting conformation for umbrella simulation, the conformations was restrained by a harmonic potential of 5000 kJ mol^−1^ nm^−1^ applied between the center of mass of two equally size groups. Group 1 consisted of the Cα atoms of residue 11 to 20 on helix TM1A, group 2 included the Cα atoms of residues 78 to 81 (intracellular loop 1), 363 to 366 (TM8) and 503 and 504 (TM12). Each window was simulated for 25 ns. The first 5 ns were discarded as equilibration, the remaining 20 ns were considered for determination of the PMF using the weighted histogram analysis method (WHAM) [58]. Statistical errors were estimated using the bootstrap method.

### Protein construction

The LBT tag has the amino acid sequence YWDTNNDGWYEGDELLA. The LBT encoded at the DNA level, was introduced just after the C-terminus to the LeuT gene within thrombin cleavage site (LVPAGS) right before the HIS-tag using two consecutive PCRs. The plasmid for LeuT was a kind gift by E. Gouaux. First PCR reaction was carried out to make the megaprimers using primers (R519-LBT-G520 primers), and purified using agarose gel. These cleaned megaprimers were then used to introduce the LBT encoding peptide at the C-terminus of the LeuT gene via conventional PCR yielding the LeuT-LBT construct. The A9C mutation was introduced via site directed mutagenesis into the LeuT-LBT construct using respective primers. All mutations were confirmed via DNA sequencing. Below, the sequence of the LBT tag is highlighted in bold.

Protein sequence of LeuT-LBT:

MEVKREHWATRLGLILAMAGNAVGLGNFLRFPVQAAENGGGAFMIPYIIAFLLVGIPLMWIEWAMGRYGGAQGHGTTPAIFYLLWRNRFAKILGVFGLWIPLVVAIYYVYIESWTLGFAIKFLVGLVPEPPPNATDPDSILRPFKEFLYSYIGVPKGDEPILKPSLFAYIVFLITMFINVSILIRGISKGIERFAKIAMPTLFILAVFLVIRVFLLETPNGTAADGLNFLWTPDFEKLKDPGVWIAAVGQIFFTLSLGFGAIITYASYVRKDQDIVLSGLTAATLNEKAEVILGGSISIPAAVAFFGVANAVAIAKAGAFNLGFITLPAIFSQTAGGTFLGFLWFFLLFFAGLTSSIAIMQPMIAFLEDELKLSRKHAVLWTAAIVFFSAHLVMFLNKSLDEMDFWAGTIGVVFFGLTELIIFFWIFGADKAWEEINRGGIIKVPRIYYYVMRYITPAFLAVLLVVWAREYIPKIMEETHWTVWITRFYIIGLFLFLTFLVFLAERRRNHESAGTLVPR**YWDTNNDGWYEGDELLA**GSGHHHHHHHH

Protein sequence of LeuT-LBT-A9C:

MEVKREHWCTRLGLILAMAGNAVGLGNFLRFPVQAAENGGGAFMIPYIIAFLLVGIPLMWIEWAMGRYGGAQGHGTTPAIFYLLWRNRFAKILGVFGLWIPLVVAIYYVYIESWTLGFAIKFLVGLVPEPPPNATDPDSILRPFKEFLYSYIGVPKGDEPILKPSLFAYIVFLITMFINVSILIRGISKGIERFAKIAMPTLFILAVFLVIRVFLLETPNGTAADGLNFLWTPDFEKLKDPGVWIAAVGQIFFTLSLGFGAIITYASYVRKDQDIVLSGLTAATLNEKAEVILGGSISIPAAVAFFGVANAVAIAKAGAFNLGFITLPAIFSQTAGGTFLGFLWFFLLFFAGLTSSIAIMQPMIAFLEDELKLSRKHAVLWTAAIVFFSAHLVMFLNKSLDEMDFWAGTIGVVFFGLTELIIFFWIFGADKAWEEINRGGIIKVPRIYYYVMRYITPAFLAVLLVVWAREYIPKIMEETHWTVWITRFYIIGLFLFLTFLVFLAERRRNHESAGTLVPR**YWDTNNDGWYEGDELLA**GSGHHHHHHHH

Primers for LBT coding Megaprimers

LeuT-LBT forward primer: AGAGTGCTGGTACCCTGGTGCCGCGCTATTGGATACCAACAACG

LeuT-LBT forward primer: TGGTGATGATGACCGCTGCCCGCCAGCAGTTCATCGCC

A9C mutant forward primer: GAAGTTAAAAGGGAACACTGGTGCACGCGACTCGGTTTAATCCTC

A9C mutant reverse primer: GAGGATTAAACCGAGTCGCGTGCACCAGTGTTCCCTTTTAACTTC

### Protein purifications and labelling

LeuT was expressed and purified as previously reported [4]. Briefly, fresh bacterial transformants of chemically competent cells C41 (DE3) from Lucigen were used for each batch of protein purification. Cells were induced with 0.2 mM IPTG when inoculum reached an OD_600_ of 0.6. Induced cells were grown for 20 hours at 20°C. Harvested cells were lysed two times on Avestin Emulsiflex applying a pressure of 15000 psi in lysis buffer (50 mM HEPES, pH 7.5, 200 mM NaCl, 1 mM EDTA, 5 mM MgCl2, 20 μg/ml DNAse-1, 1 mM PMSF and 0.4 mg/ml Lysozyme). Cell debris were removed by centrifugation at 5000g. Supernatant was centrifuged at 120000g for two hours to pellet the crude membrane fraction. Subsequently, crude membranes were solubilized for 90 min on a rotatory carousel at 4°C in membrane solubilization buffer (20 mM HEPES, pH 7.5, 200 mM NaCl and 1% DDM supplemented with 1 mM PMSF). Unsolubilized membranes were removed by centrifugation at 120000g for 20 min at 4°C. Solubilized material was incubated with pre-equilibrated NiNTA beads from Qiagen overnight at 4°C. Leucine bound to LeuT was removed by extensive washing with buffer devoid of Na^+^ (20 mM HEPES, pH 7.5, 200 mM KCl) supplemented with concentration gradient of imidazole. Bound LeuT was then eluted from the NiNTA resin with elution buffer (20 mM HEPES, pH 7.5, 200 mM KCl, 250 mM imidazole). Imidazole was then removed using PD10 columns and ion exchange between Na^+^ and K^+^ was performed as needed.

Cysteine specific protein labeling with the fluorescence dye BODIPY-C3-M was carried out using a protein to BODIPY-C3-M mole ratio of 1:3, incubated for 3 hours at 4°C by gentle agitation before LeuT was eluted from the NiNTA resin. Excessive dye was removed by washing the LeuT loaded Ni-NTA beads on a column. LRET based distance measurements were then carried out using freshly purified samples.

### Protein reconstitution

POPC proteoliposomes were prepared as reported [59] from detergent solubilized and BODIPY-C3-M labeled LeuT using a protein:lipid (w:w) ratio of 1:100. In brief POPC lipid (Avanti Polar lipids Inc.) was dried under a gentle stream of nitrogen to remove the organic solvent chloroform, remaining chloroform traces were removed overnight in a rotavapor. The lipid film was dissolved in 200 mM KCl or NaCl buffer and 20 mM HEPES at pH 7.5 to a final concentration of 20 mg/ml. The lipid suspension was then sonicated for 45 minutes (using three 15-min cycles), flash frozen and slowly thawed to room temperature. Liposomes were extruded 11 times with a mini extruder (Avanti lipids) over a filter of pore size of 400 nm. Liposomes were destabilized with Triton X-100 and detergent solubilized LeuT added at a 1:100 protein:lipid ratio and incubated at room temperature for 30 min. Detergent was removed using biobeads (Biorad) followed by ultracentrifugation at 120000g for 90 min. The proteoliposomes were re-suspended to a final concentration of 100 mg/ml and stored at -80°C until use.

### Synthesis of BODIPY-C3-M (1)

**Fig 7:**
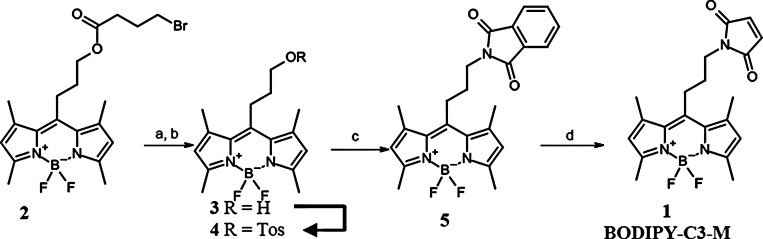
Synthesis scheme for BODIPY-C3-M. a) MeOH/DCM (2:1), LiOH (aq), rt, 4h; b) DCM, tosyl chloride, NEt_3_, DMAP 0°C, rt; c) Phthtalimide potassium salt, MeCN (5% DMSO), 50°C, 24 h; d) 1. Hydrazine, EtOH/DCM; 2. DCM, *N*-methoxycarbonylmaleimide, rt, 52 h.

First LRET measurements performed with a commercially available BODIPY-based acceptor fluorophore did not meet with any success. This was thought to be due to the extended length of the spacer of this probe joining the BODIPY dye with the thiol reactive maleimide subunit as well as to the nonsymmetrical attachment of the linker to the fluorescent moiety, both counteracting a well-defined spatial orientation of the probe in the protein. To overcome this problem the BODIPY probe **1** (BODIPY-C3-M, Fig 7) has been developed in which a short three carbon chain symmetrically originates from the BODIPY dye and links it with the maleimide moiety.

### Synthesis strategy

As starting material for the synthesis of BODIPY-C3-maleimide probe **1**, a BODIPY derivative analogous to **2** but equipped with a 3-bromopropyl residue in 10-position should be used. But when following a standard procedure [60] common for the preparation of 10-substituted BODIPY derivatives starting from 2,4-dimethylpyrrole and 4-bromobutyryl chloride, instead of the desired BODIPY derivative displaying a 3-bromopropyl residue, the BODIPY derivative **2** was obtained. As even upon extensive variation of the reaction conditions only **2** could be isolated, the formation of which was later on also published by others in the literature [61], this compound, **2**, was used as starting material for the preparation of the target BODIPY **1**. To this end, compound **2** was subjected to an alkaline hydrolysis of the ester function to yield alcohol **3** which was subsequently transformed in the O-tosylated derivative **4** upon treatment with tosyl chloride in the presence of NEt_3_ and DMAP. Reaction of **4** with the potassium salt of phthalimide gave BODIPY derivative **5**. Subsequent treatment of **5** with hydrazine to liberate the primary amino function contained in **5** and with N-methoxycarbonylmaleimide without prior isolation of the amino derivative finally provided BODIPY probe **1.**

#### Synthesis of 10-{3-[(4-Bromobutanoyl)oxy]propyl}-5,5-difluoro-1,3,7,9-tetramethyl-5H-dipyrrolo[1,2-c:2',1'-f][1,3,2]diazaborinin-4-ium-5-uide (2)

The synthesis has been accomplished in analogy to a literature procedure [60] by reacting 2,4-dimethylpyrrole (2.28 g, 24.0 mmol, 2.47 mL) in CH_2_Cl_2_ (135 mL) with 4-bromobutyryl chloride (2.22 g, 12.0 mmol, 1.38 mL) and by treating the residue resulting from evaporation of the solvent in vacuo with NEt_3_ (5.83 g, 57.6 mmol, 7.98 mL) and BF_3_•OEt_2_ (11.80 g, 83.14 mmol, 10.53 mL) in toluene and CH_2_Cl_2_ (300 mL, 95:5). Purification by flash column chromatography (silica gel; pentane/EtOAc = 4:1) yielded **2** as orange solid (2.07 g, 76%). Analytical data were in accord with those later reported in literature [61].

#### Synthesis of 5,5-Difluoro-10-(3-hydroxypropyl)-1,3,7,9-tetramethyl-5H-dipyrrolo[1,2-c:2',1'-f][1,3,2]diazaborinin-4-ium-5-uide (3)

To **2** (1.88 g, 4.13 mmol) dissolved in CH_2_Cl_2_ and CH_3_OH (2:1, 60 ml), a 0.991 M solution of lithium hydroxide in H_2_O (10 mL) was added dropwise. The reaction was stirred for 4 h at room temperature. After removing of the solvent in vacuo, the remaining residue was dissolved in EtOAc and washed with H_2_O, brine, and again with H_2_O. The organic phase was dried with Na_2_SO_4_, filtered, and concentrated in vacuo to yield **3** as orange solid (1.21 g, 96%). Mp 188–190°C. IR (KBr): υ = 3560, 3374, 2954, 2869, 1549, 1508, 1473, 1409, 1368, 1305, 1268, 1225, 1202, 1161, 1134, 1102, 1078, 1056 cm^-1^. ^1^H NMR (400 MHz, CD_2_Cl_2_, TMS) *δ* = 1.62 (t, *J* = 4.9 Hz, 1 H), 1.77–1.90 (m, 2 H), 2.44 (s, 6 H), 2.47 (s, 6 H), 3.02–3.09 (m, 2 H), 3.77 (dd, *J* = 10.9/5.7 Hz, 2 H), 6.09 (s, 2 H). ^13^C NMR (100 MHz, CD_2_Cl_2_, TMS) *δ* = 14.56 (2 C), 14.59 (2 C), 16.66, 25.47, 34.83, 62.73, 121.98 (2 C), 131.76, 141.29 (2 C), 146.94, 154.19 ppm. ^19^F NMR (470 MHz, CD_2_Cl_2_) *δ* = -146.43 (dd, *J* = 32.63, 66.27 Hz,) ppm.^11^B NMR (160 MHz, CD_2_Cl_2_) *δ* = -2.25 (t, *J* = 32.98 Hz, H) ppm. MS (CI, CH_5_^+^) m/z (%): 307.15 (18, [M+H]^+^), 287.15 (100). HRMS (EI+): M^+^ calc. for C_16_H_21_BF_2_N_2_O, 306.1715; found: 306.1718.

#### Synthesis of 5,5-Difluoro-1,3,7,9-tetramethyl-10-[3-(p-tolylsulfonyloxy)propyl]-5H-dipyrrolo[1,2-c:2',1'-f][1,3,2]diazaborinin-4-ium-5-uide (4)

To **3** (1.38 g, 4.51 mmol) in CH_2_Cl_2_ (45 ml) at 0°C, NEt_3_ (1.46 g, 14.4 mmol, 2.00 ml), DMAP (55 mg, 0.45 mmol), and TosCl (1.72 g, 9.05 mmol) were added. The reaction mixture was allowed to warm up to room temperature and stirred overnight. Then, it was washed with H_2_O, brine, and H_2_O and the organic phase was dried with Na_2_SO_4_, filtered and concentrated in vacuo. The product was purified by flash column chromatography (silica gel; CH_2_Cl_2_/isohexane = 3:1) to yield an orange solid (1.94 g, 94%). Mp 170.5–172.5°C.R_f_ = 0.3. IR (KBr): ν = 2960, 2924, 1598, 1552, 1512, 1473, 1408, 1361, 1307, 1277, 1224, 1203, 1176, 1159, 1096, 1077, 1026 cm^-1^. ^1^H NMR (400 MHz, CD_2_Cl_2_, TMS) *δ* = 1.94 (dt, *J* = 17.2/5.8 Hz, 2 H), 2.36 (s, 6 H), 2.45 (s, 9 H), 2.96–3.02 (m, 2 H), 4.16 (t, *J* = 5.9 Hz, 2 H), 6.08 (s, 2 H), 7.39 (d, *J* = 7.9 Hz, 2 H), 7.78 (d, *J* = 8.3 Hz, 2 H) ppm. ^13^C NMR (100 MHz, CD_2_Cl_2_, TMS) *δ* = 14.63, 16.59, 21.82, 25.03, 31.15, 70.10, 122.18 (2 C), 128.17, 130.40, 131.62 (2 C), 133.13, 141.10, 144.75, 145.76, 154.69 ppm. ^19^F NMR (470 MHz, CD_2_Cl_2_) *δ* = -146.48 (dd, *J* = 30.91, 64.77 Hz) ppm. ^11^B NMR (160 MHz, CD_2_Cl_2_) *δ* = -2.34 (t, *J* = 32.84 Hz) ppm. MS (CI, CH_5_^+^) m/z (%): 461.12 (12, [M+H]^+^), 442.20 (31), 441.20 (100). HRMS (EI+): M^+^ calc. for C_23_H_27_BF_2_N_2_O_3_S, 460.1804; found: 460.1821.

#### Synthesis of 10-[3-(1,3-Dioxo-2,3-dihydro-1H-isoindolin-2-yl)propyl]-5,5-difluoro-1,3,7,9-tetramethyl-5H-dipyrrolo[1,2-c:2',1'-f][1,3,2]diazaborinin-4-ium-5-uide (5)

To a solution of **4** (1.08 g, 2.34 mmol) in MeCN (25 ml, 5% DMSO), phthalimide potassium salt (663 mg, 3.51 mmol) was added and the reaction mixture was stirred for 24 h at 50°C. Then, the reaction mixture was filtered, the solvent removed in vacuo, and the product purified by flash column chromatography (silica gel; CHCl_3_/EtOAc = 98:2) to yield an orange solid (143 mg, 14%). Mp 177.6–186.9°C.R_f_ = 0.37. IR (KBr): υ = 3463, 1930, 1773, 1714, 1616, 1548, 1508, 1466, 1432, 1398, 1368, 1335, 1304, 1280, 1225, 1195, 1161, 1089, 1077, 1059, 1028 cm^-1^. ^1^H NMR (500 MHz, CDCl_3_, TMS) *δ* = 1.95–2.05 (m, 2 H), 2.41 (s, 6 H), 2.50 (s, 6 H), 2.98–3.05 (m, 2 H), 3.86 (t, *J* = 7.3 Hz, 2 H), 6.04 (s, 2 H), 7.74 (dd, *J* = 5.5/3.0 Hz, 2 H), 7.86 (dd, *J* = 5.4/3.1 Hz, 2 H) ppm. ^13^C NMR (125 MHz, CDCl_3_) *δ* = 14.60 (2 C), 16.39 (2 C), 25.95, 30.52, 37.88, 121.95 (2 C), 123.52, 131.44, 132.02 (2 C), 134.34 (2 C), 140.44 (2 C), 144.48, 154.39 (2 C), 168.36 (2 C) ppm. ^11^B NMR (160 MHz, CDCl_3_) *δ* = -0.44 (t, *J* = 32.93 Hz) ppm. ^19^F NMR (470 MHz, CDCl_3_) *δ* = -146.55 (dd, *J* = 32.11/65.60) ppm. MS (EI) m/z (%): 435.33 (M^+^, 100), 262.24 (74), 255.14 (90). HRMS (EI): M^+^ calc. for C_24_H_24_BF_2_N_3_O_2_ 435.1930; found: 435.1924.

#### Synthesis of 10-[3-(2,5-Dioxo-2,5-dihydro-1*H*-pyrrol-1-yl)propyl]-5,5-difluoro-1,3,7,9-tetramethyl-5*H*-dipyrrolo[1,2-*c*:2',1'-*f*][1,3,2]diazaborinin-4-ium-5-uide (1)

To a solution of **5** (224 mg, 515 μmol) in CH_2_Cl_2_/EtOH (1:1, 20 ml), hydrazine hydrate (404 mg, 5.17 mmol, 393 μl) was added dropwise. The reaction mixture was stirred for 18 h at room temperature, filtered, and concentrated in vacuo. The resulting residue was treated with cold CH_2_Cl_2_, filtered to remove any remaining white solid and the CH_2_Cl_2_ phase was concentrated. The resulting residue was repeatedly treated as described before, until no additional precipitation of white solid occurred. The final residue (146 mg, 0.48 mmol) was dissolved in CH_2_Cl_2_ (65 mL), DIPEA (145 mg, 1.15 mmol, 191 μl), and N-methoxycarbonylmaleimide (148 mg, 954 μmol) were added and the reaction mixture was stirred for 52 h at room temperature. The solvent was removed in vacuo and the product was purified by flash column chromatography (silica gel; CH_2_Cl_2_) to yield 1 as orange solid (126 mg, 68%). Mp 237.6–239.2°C. R_f_ = 0.35. IR (KBr): υ = 3453, 3118, 3**1**04, 2962, 2934, 2872, 1740, 1704, 1552, 1509, 1475, 1448, 1438, 1409, 1378, 1366, 1337, 1309, 1284, 1225, 1204, 1154, 1117, 1103, 1080, 1062, 1047 cm^-1^. ^1^H NMR (400 MHz, CDCl_3_) *δ* = 1.83–1.97 (m, 2 H), 2.40 (s, 6 H), 2.50 (s, 6 H,), 2.90–2.99 (m, 2 H), 3.69 (t, *J* = 7.2 Hz, 2 H), 6.05 (s, 2 H), 6.74 (s, 2 H). ^13^C NMR (100 MHz, CDCl_3_) *δ* = 14.60 (2 C) 16.41 (2 C), 25.82, 30.53, 37.74, 121.97 (2 C), 131.38 (2 C), 134.33 (2 C), 140.35 (2 C), 144.37, 154.43 (2 C), 170.67 (2 C). ^19^F NMR (471 MHz, CDCl_3_) *δ* = -146.52 (ddd, *J* = 9.46, 32.70, 43.16 Hz). ^11^B NMR (160 MHz, CDCl_3_) *δ* = -0.44 (t, *J* = 32.90 Hz, H). MS (EI, 70 eV) m/z (%): 385.13 (100, M^+^), 262.08 (64). HRMS (EI): M^+^ calc. for C_20_H_22_BF_2_N_3_O_2_, 385.1773; found: 385.1773.

### LRET measurements

The D535-30 bandpass filter (Chroma) was used for the measurements of the donor-emission (viz. Tb^3+^ emission). The transmission range of this filter is centered on the second emission peak of Tb^3+^ at the wavelength of 540 nm. BODIPY-C3-M (acceptor) emission was recorded in the dark region between the first and the second Tb^3+^ peak by utilizing a filter specified for this wavelength range: D520/25m (Chroma). However, we found poor signal to noise ratios in our recordings of the acceptor emission. We surmise that this was likely caused by quenching of the acceptor fluorophore emission by the environment. However, quenching of the acceptor emission has no repercussion on the Förster-distance. Accordingly, all analyses presented here are based on the parameters estimated from the fits to the donor decays in the absence and presence of the acceptor.

We gated the photomultiplier tube at 300 μs after triggering of the laser pulse and fitted the donor emission by a sum of two exponentials. The faster component of around 400 μs was present in all conditions (also in the fluorophore free condition) indicating that this component is a non-specific component and independent of fluorophore absorption and emission (S1 Table). Samples containing 1 μM detergent micelle solubilized LeuT in HEPES buffer, NaCL or KCL 200 mM, and 2 μM TbCL_3_ at pH 7.5 were placed onto the fine quartz coverslip. Liposome reconstituted LeuT samples were prepared containing 5 μM protein. HEPES buffer, NaCl or KCl 200 mM at pH 7.5 were the same outside and inside the liposomes. 10 μM TbCl_3_ was added to the outside of the liposomes. LBT-tag bound Tb^3+^ was excited by the UV laser pulse with a wavelength of 266 nm, fluorescence decay traces were recorded. Decay traces were fitted as shown in supplementary table and distances between LBT-tag bound Tb^3+^ and acceptor fluorophore were calculated from the decay constants τ_DA_ using the Förster relationship [62].

$$R=R_{0}\;*\;{\left(\frac{\tau_{DA}}{\tau_{D}-\tau_{DA}}\right)}^{1/6}$$ Equation 1

R is the calculated donor-acceptor distance, R_0_ is the reference distance, τ_D_ is the intrinsic donor decay, τ_DA_ is the sensitized donor decay constant due to excitation energy transfer from the donor to the acceptor.

### [^3^H]-Ala uptake

[^3^H]-Ala uptake was carried out at 30°C as reported previously [63]. Proteoliposomes were prepared as described above. The internal solution of proteoliposomes contained 200 mM KCl, 20 mM HEPES at pH 7.5. Background binding was determined using gradient free proteoliposomes containing 200 mM NaCl instead of KCl. The uptake was initialized by diluting the proteoliposomes into external buffer (200 mM NaCl, 20 mM HEPES at pH 7.5, at indicated concentrations of [^3^H]-Ala) at 30°C. The reaction was stopped after two minutes by 10 folds dilution into ice cold stopping buffer (200 mMKCl, 20 mM HEPES at pH 7.5). Proteoliposomes were then collected over nitrocellulose filters of 0.22 μm pore size. The filters were washed three times with 4 mL of ice-cold stopping buffer. The dried filters were immersed into 3 ml of scintillation cocktail. Radioactivity was measured in a liquid scintillation counter after mixing on a shaker for 2 hours.

## Supporting Information

S1 Fig Overall stability of the simulations. RMSD plot shows the deviation of the Cα atoms with respect to the starting structure for (**A**) the outward-occluded, (**B**) the inward-open and (**C**) the micelle simulations. The RMSD initially increases as expected and then levels off a value of ~0.2 nm, indicative of stable simulations with a small overall structural deviation from the starting coordinates. The drift, that is visible in the simulations of the inward-open membrane inserted LeuT correlates with the movement of TM1A.
(TIF)

S2 Fig Vestibule size. The radius of the intracellular vestibule was measured using the caver 3.0 program for the **(A)** crystal structure (PDB ID: 3TT3), the final structures of the MD simulations of membrane inserted LeuT, **(B)** run1, **(C)** run2, and **(D)** run3, and the final structure of the MD simulations of micelle inserted LeuT, **(F)** run1, **(G)** run2, and **(H)** run3. The protein is shown in simplified trace configuration, while TM1A is highlighted in ribbon representation. The size of the vestibule was determined by the program caver 3.0 and is show in blue. The size of the vestibule was quantified and shown in (E) for membrane and (I) for micelle systems. The x-axis represents the distance along the vestibule starting from the S1 substrate binding site, the radius shows the size of the vestibule. The inner vestibule remained open in both the micelle and the membrane inserted system.
(TIF)

S3 Fig Partitioning of the side chain of residue R11. The number of water molecules and lipid phosphate (PO_4_^-^) groups within 0.5 nm of the guanidinium group of residue R11 are shown for (**A**) the outward-occluded simulations and (**B**) the inward-open system. The number of interactions increases over time in **B**, indicating increasing exposure to the hydrophilic environment, which correlates with the conformational change of TM1A. (**C**) Number of water molecules within 0.5 nm of the guanidinium group of R11 in the micelle systems.
(TIF)

S4 Fig Membrane thickness of the inward-open LeuT simulations. **(A)** View to LeuT from the cytosolic site: TM1A is highlighted in purple, the membrane in tan. All systems were oriented by fitting to LeuT as shown in panel A. Membrane thickness is averaged over the first 50 ns (run1 **B**, run2 **D**, and run3 **F**) and over the last 50 ns (run1 **C**, run2 **E**, and run3 **G**). LeuT is not shown in panel B-G for clarity. Membrane thickness is color coded using the scale shown in the legend on the right. The same scale was used for in all panels. Membrane thickness was increased next to TM1A in the beginning of the simulations, while TM1A was still within the membrane core. Deviations from the average thickness were less pronounces towards the end of the simulations, triggered by re-partitioning of TM1A. It is interesting to note that two lipid molecule interacting with TM1A were elevated above the membrane, resulting in a large local increase in membrane thickness, clearly visible as the red colored area in panel E.
(TIF)

S5 Fig Dissociation of Na2. Quantification of the movement of Na2 away from its initial position in the simulations of the membrane embedded LeuT. (**A**) Na2 remains stably bound to the outward-occluded conformation of LeuT throughout the simulations. (**B**) Na2 dissociates from the inward-open conformation of LeuT within the first 5 ns.
(TIF)

S6 Fig Substrate uptake into proteoliposomes. Uptake of ^3^H-Ala into POPC proteoliposomes was performed over 2 min in the presence of increasing concentrations of substrate. Wild type LeuT (black), LeuT-LBT (green) and the LeuT-LBT-A9C (purple) construct showed indistinguishable uptake kinetics. Data are shown from three independent experiments performed in duplicates, error bars denote S.E.M.
(TIF)

S7 Fig Simulation box. Representative final structures for each system are shown for (**A**) the outward-occluded, (**B**) the inward-open system and (**C**) the micelle system containing 140 BOG molecules. LeuT is shown in green ribbon representation, bound sodium ions as green spheres, substrate leucine as green sticks, membrane (POPC) in gray, phosphate atoms as dark spheres, BOG detergent in yellow, the O1 atoms BOG as orange sphere, sodium ions as blue sphere, chloride ions as pink spheres, and water as red-white sticks.
(TIF)

S1 Table Fit-parameters for the LRET donor decay. Donor emission decays were fit to a sum of two exponentials. The table shows the estimated τ values for the two components along with their respective fractional amplitudes. Each value is the mean of three independent experiments performed in triplicates ± SEM.
(PDF)
