# Supplementary figures and images for: The Environment Shapes the Inner Vestibule of LeuT
Source: PLoS Comput Biol. 2016 Nov 11;12(11):e1005197. doi: 10.1371/journal.pcbi.1005197 (PMC5105988; doi:10.1371/journal.pcbi.1005197)

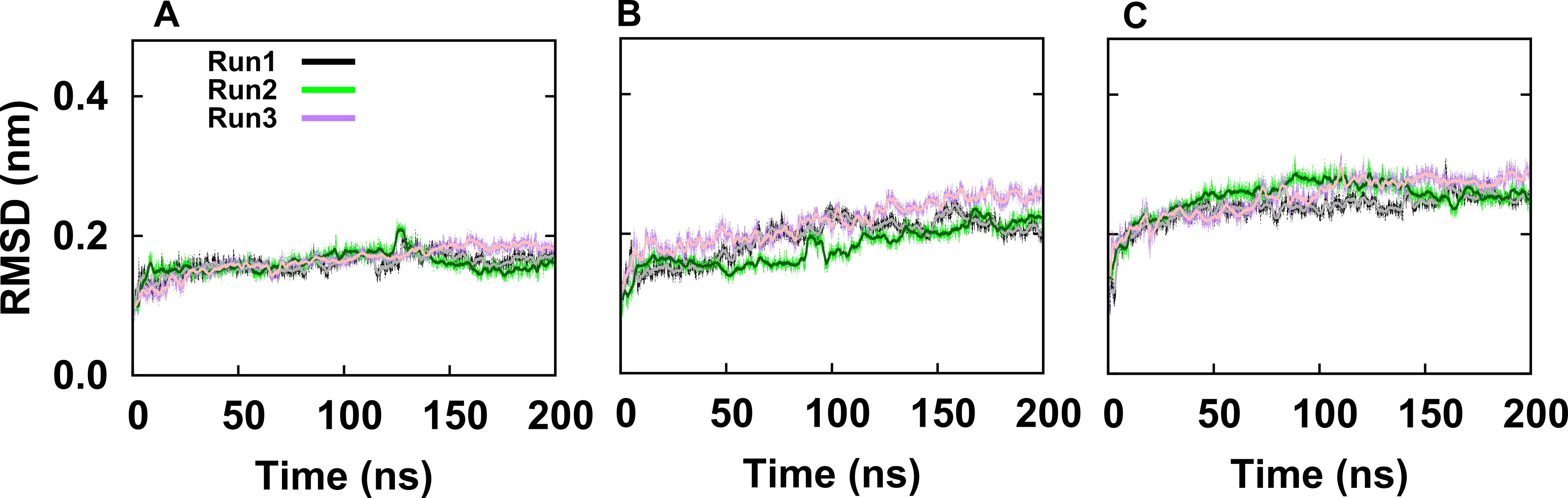

Supplement: S1 Fig — RMSD plot shows the deviation of the Cα atoms with respect to the starting structure for (A) the outward-occluded, (B) the inward-open and (C) the micelle simulations. The RMSD initially increases as expected and then levels off a value of ~0.2 nm, indicative of stable simulations with a small overall structural deviation from the starting coordinates. The drift, that is visible in the simulations of the inward-open membrane inserted LeuT correlates with the movement of TM1A. (TIF) [file pcbi.1005197.s001.tif]

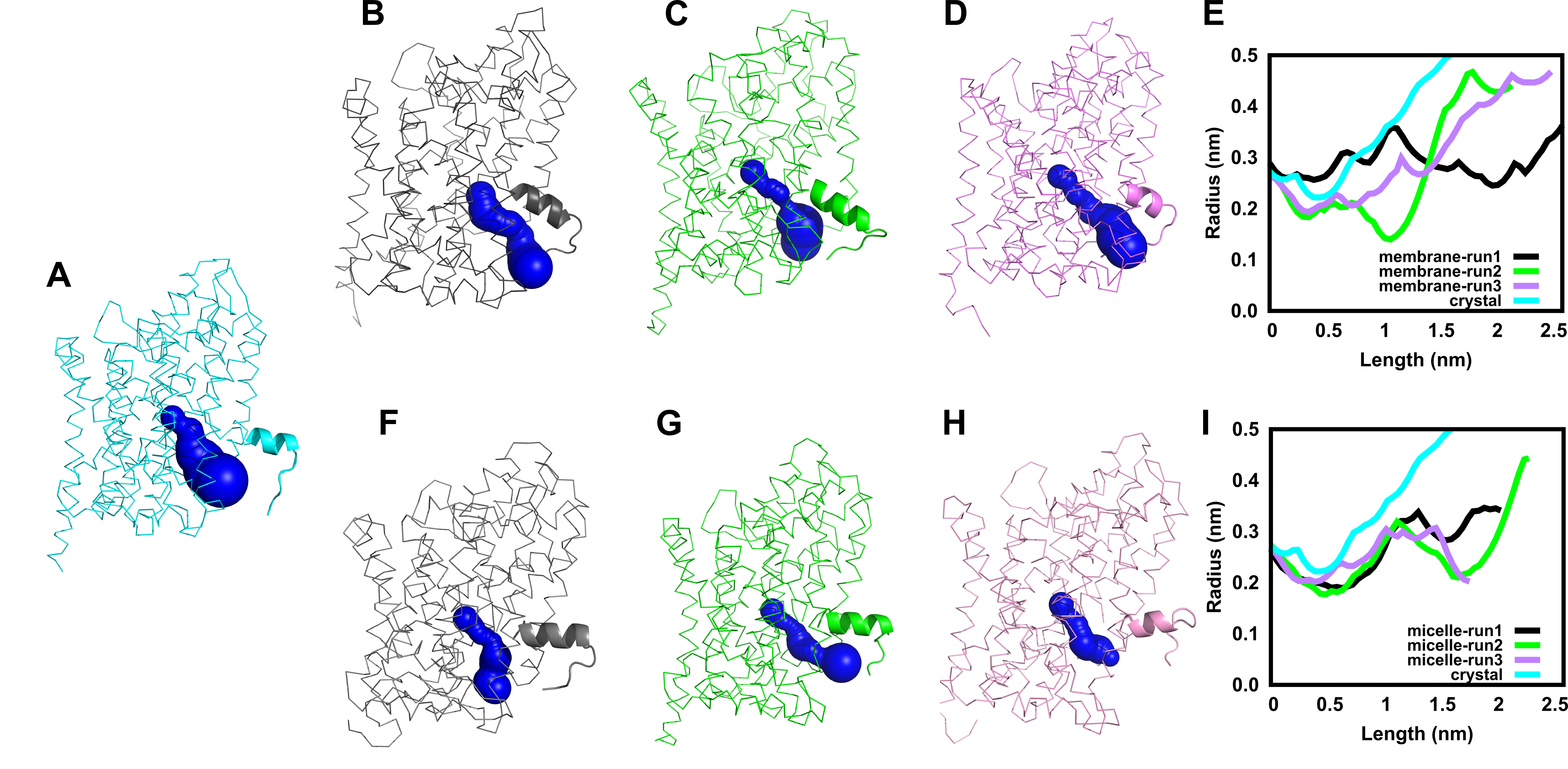

Supplement: S2 Fig — The radius of the intracellular vestibule was measured using the caver 3.0 program for the (A) crystal structure (PDB ID: 3TT3), the final structures of the MD simulations of membrane inserted LeuT, (B) run1, (C) run2, and (D) run3, and the final structure of the MD simulations of micelle inserted LeuT, (F) run1, (G) run2, and (H) run3. The protein is shown in simplified trace configuration, while TM1A is highlighted in ribbon representation. The size of the vestibule was determined by the program caver 3.0 and is show in blue. The size of the vestibule was quantified and shown in (E) for membrane and (I) for micelle systems. The x-axis represents the distance along the vestibule starting from the S1 substrate binding site, the radius shows the size of the vestibule. The inner vestibule remained open in both the micelle and the membrane inserted system. (TIF) [file pcbi.1005197.s002.tif]

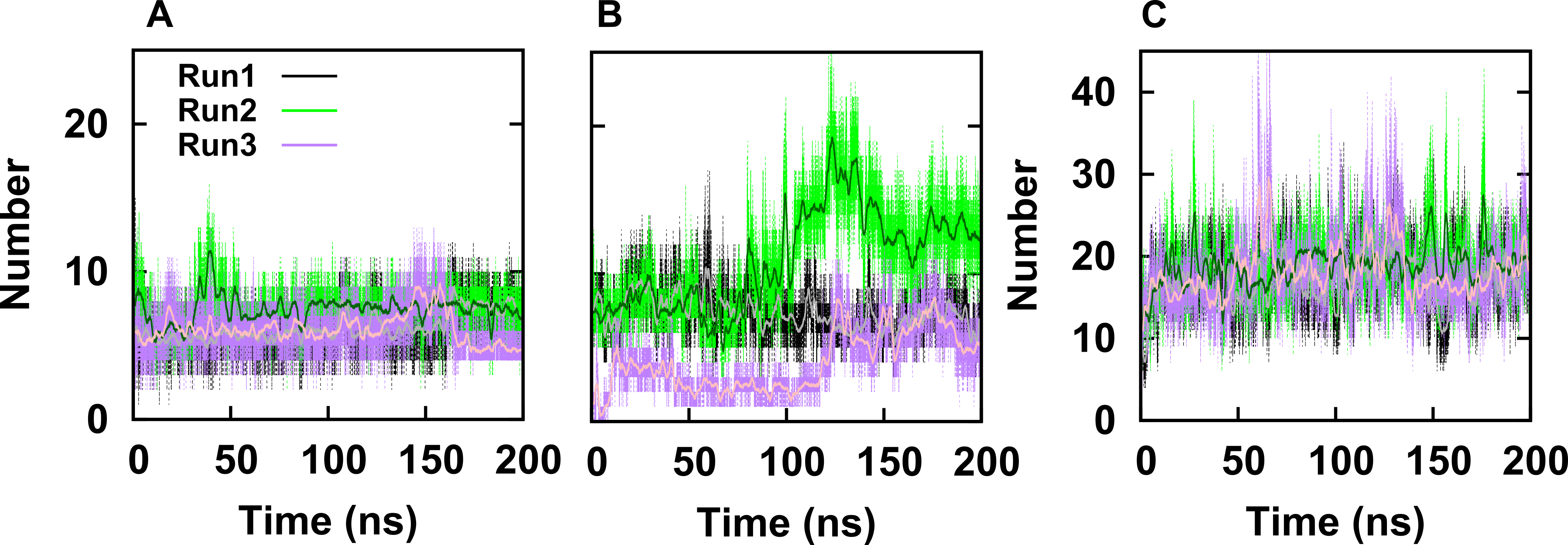

Supplement: S3 Fig — The number of water molecules and lipid phosphate (PO4-) groups within 0.5 nm of the guanidinium group of residue R11 are shown for (A) the outward-occluded simulations and (B) the inward-open system. The number of interactions increases over time in B, indicating increasing exposure to the hydrophilic environment, which correlates with the conformational change of TM1A. (C) Number of water molecules within 0.5 nm of the guanidinium group of R11 in the micelle systems. (TIF) [file pcbi.1005197.s003.tif]

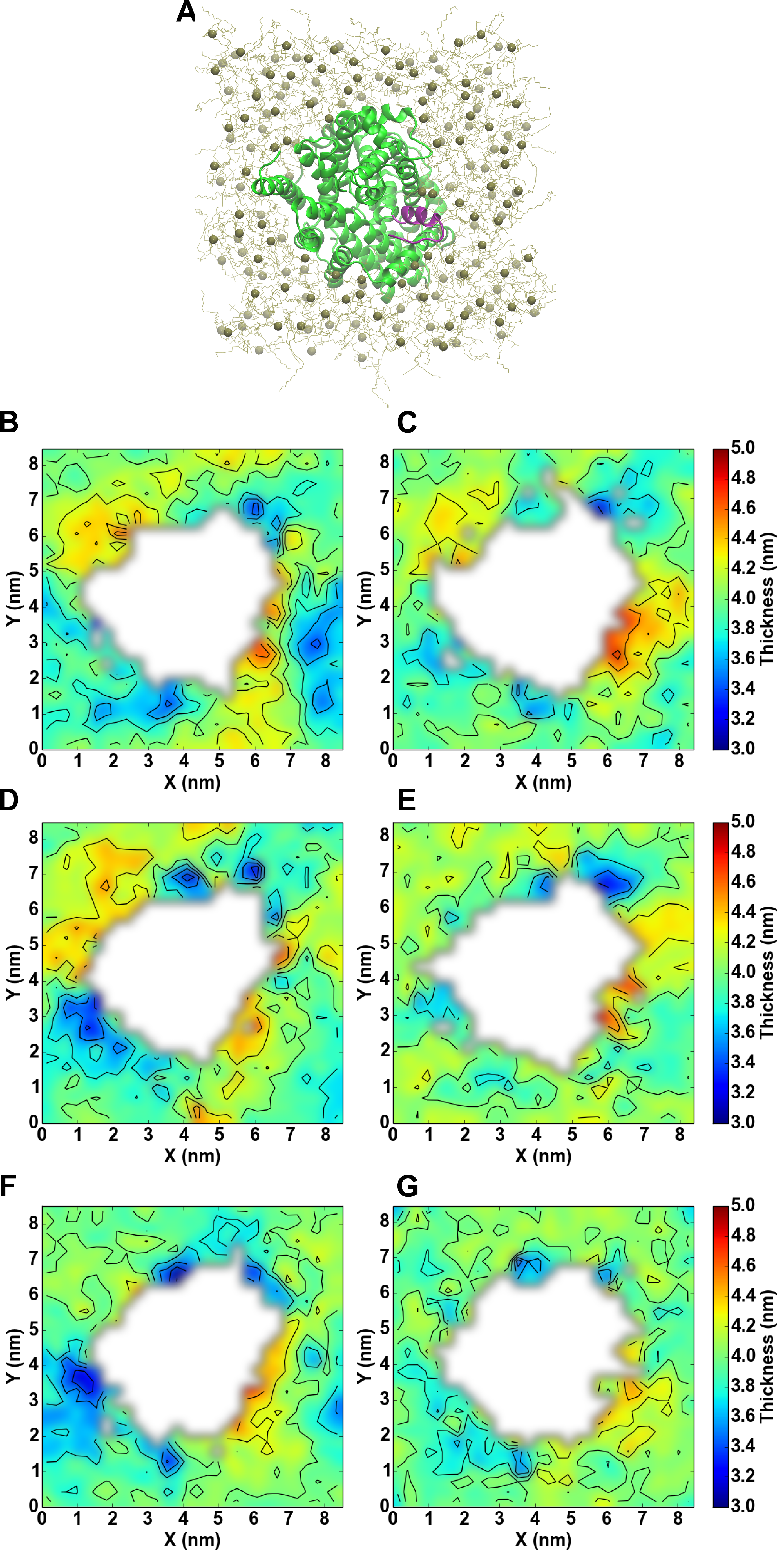

Supplement: S4 Fig — (A) View to LeuT from the cytosolic site: TM1A is highlighted in purple, the membrane in tan. All systems were oriented by fitting to LeuT as shown in panel A. Membrane thickness is averaged over the first 50 ns (run1 B, run2 D, and run3 F) and over the last 50 ns (run1 C, run2 E, and run3 G). LeuT is not shown in panel B-G for clarity. Membrane thickness is color coded using the scale shown in the legend on the right. The same scale was used for in all panels. Membrane thickness was increased next to TM1A in the beginning of the simulations, while TM1A was still within the membrane core. Deviations from the average thickness were less pronounces towards the end of the simulations, triggered by re-partitioning of TM1A. It is interesting to note that two lipid molecule interacting with TM1A were elevated above the membrane, resulting in a large local increase in membrane thickness, clearly visible as the red colored area in panel E. (TIF) [file pcbi.1005197.s004.tif]

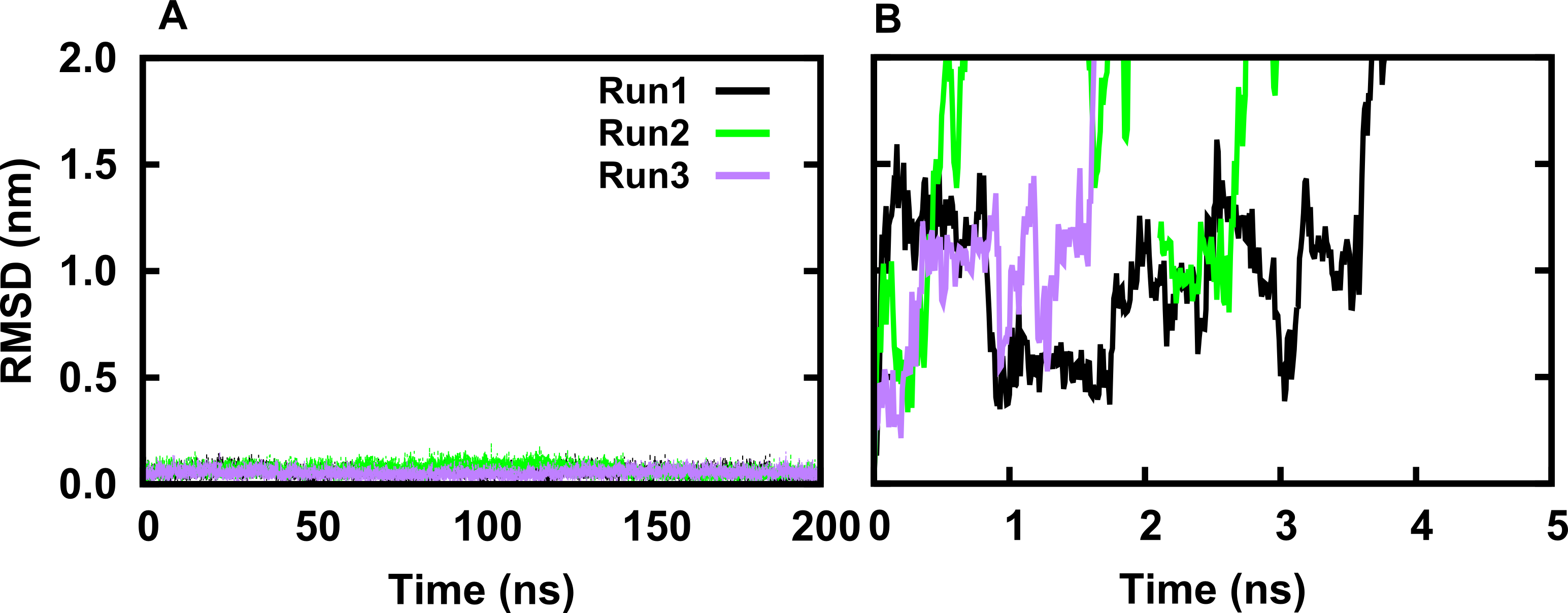

Supplement: S5 Fig — Quantification of the movement of Na2 away from its initial position in the simulations of the membrane embedded LeuT. (A) Na2 remains stably bound to the outward-occluded conformation of LeuT throughout the simulations. (B) Na2 dissociates from the inward-open conformation of LeuT within the first 5 ns. (TIF) [file pcbi.1005197.s005.tif]

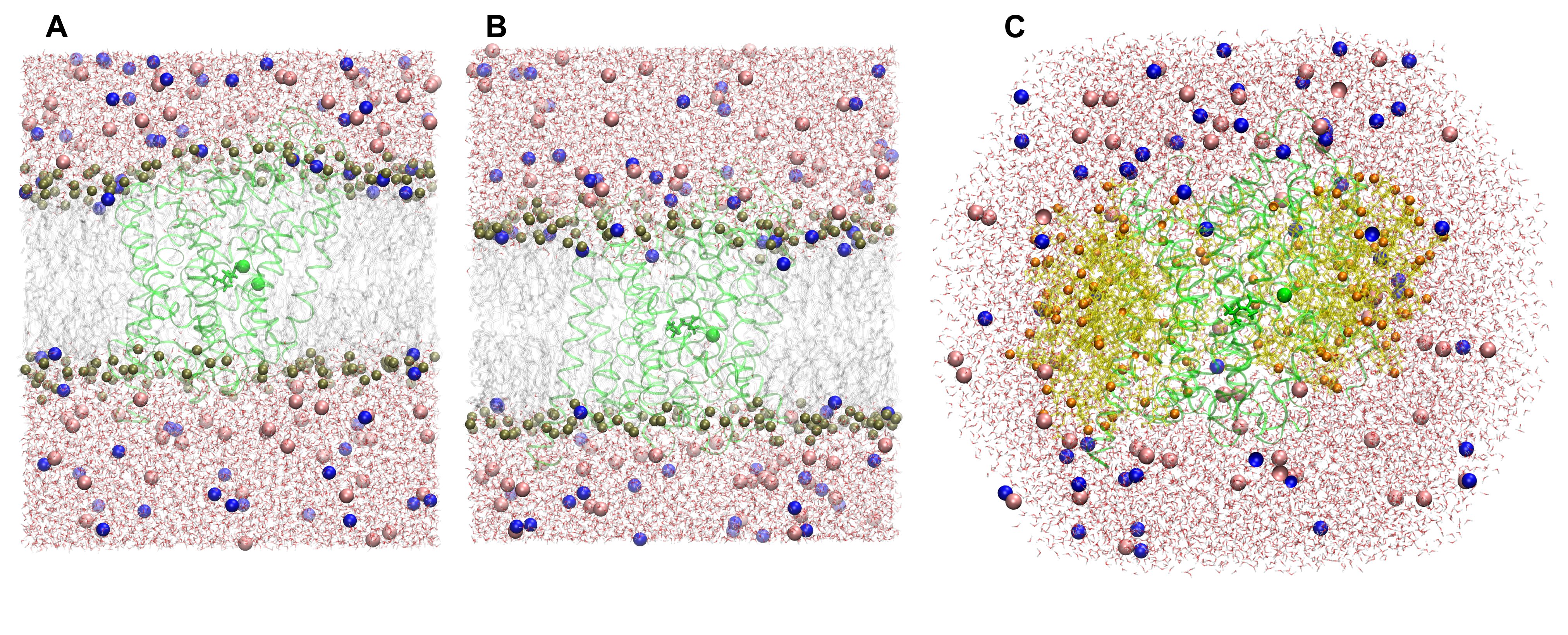

Supplement: S7 Fig — Representative final structures for each system are shown for (A) the outward-occluded, (B) the inward-open system and (C) the micelle system containing 140 BOG molecules. LeuT is shown in green ribbon representation, bound sodium ions as green spheres, substrate leucine as green sticks, membrane (POPC) in gray, phosphate atoms as dark spheres, BOG detergent in yellow, the O1 atoms BOG as orange sphere, sodium ions as blue sphere, chloride ions as pink spheres, and water as red-white sticks. (TIF) [file pcbi.1005197.s007.tif]
