# Supplementary material for: The Environment Shapes the Inner Vestibule of LeuT
Source: PLoS Comput Biol. 2016 Nov 11;12(11):e1005197. doi: 10.1371/journal.pcbi.1005197 (PMC5105988; doi:10.1371/journal.pcbi.1005197)
Supplement: S1 Table — Donor emission decays were fit to a sum of two exponentials. The table shows the estimated τ values for the two components along with their respective fractional amplitudes. Each value is the mean of three independent experiments performed in triplicates ± SEM. (PDF) [file pcbi.1005197.s008.pdf]

		Tau (τ1)	Avg. Fractions	Tau (τ2)	Avg. Fractions
**Micelles**	**Label free**	0.53 ± 0.26	0.65 ± 0.06	2.41 ± 0.01	0.35 ± 0.04
	**Label free + Na^+**	0.51 ± 0.31	0.62 ± 0.08	2.38 ± 0.06	0.38 ± 0.02
	**Labeled**	0.45 ± 0.07	0.67 ± 0.05	2.18 ± 0.02	0.37 ± 0.05
	**Label free + Na^+**	0.46 ± 0.03	0.61 ± 0.04	1.76 ± 0.08	0.39 ± 0.06
**Liposomes**	**Label free**	0.37 ± 0.14	0.70 ± 0.03	2.18 ± 0.02	0.30 ± 0.07
	**Label free + Na^+**	0.44 ± 0.17	0.74 ± 0.02	2.25 ± 0.02	0.26 ± 0.02
	**Labeled**	0.42 ± 0.04	0.69 ± 0.08	1.66 ± 0.04	0.31 ± 0.08
	**Label free + Na^+**	0.41 ± 0.04	0.71 ± 0.04	1.48 ± 0.05	0.27 ± 0.05

Table_S1 : Table of fit parameters Donor emission decays were fit to a sum of two exponentials. The table shows the estimated taus for the two components, along with their respective fractional amplitudes. Each value is the mean of three independent experiments performed in triplicates ± SEM.
